# The effects of acute high‐intensity interval exercise on the temporal dynamics of working memory and contralateral delay activity

**DOI:** 10.1111/psyp.14112

**Published:** 2022-05-30

**Authors:** Eric S. Drollette, Caroline C. Meadows

**Affiliations:** ^1^ Department of Kinesiology University of North Carolina at Greensboro Greensboro North Carolina USA

**Keywords:** contralateral delay activity, ERPs, exercise, working memory, young adults

## Abstract

The present investigation examined the acute effects of high‐intensity interval exercise (HIIE) on temporal changes in behavioral and neuroelectrical indices of working memory. Young adults (*n* = 22) performed a visual working memory change detection task of equiprobable 2‐ to 5‐dot set sizes while contralateral delay activity (CDA) and N2pc ERP components were assessed at three consecutive time periods (40‐min, 54‐min, and 68‐min) following three separate counterbalanced 9‐min sessions of seated rest, HIIE‐aerobic (treadmill intervals of moderate‐ and high‐intensity run/walk periods) and HIIE‐aerobic/resistance (intervals of rest and body‐weight calisthenics). Behavior results revealed greater 4‐dot accuracy for HIIE‐aerobic/resistance compared to seated rest only at 40‐min, maintenance of 5‐dot accuracy across time for HIIE‐aerobic compared to HIIE‐aerobic/resistance and seated rest, and greater temporal stability in overall accuracy performance (i.e., inter‐class correlation between temporally adjacent assessments) for both HIIE conditions compared to seated rest. CDA and N2pc results revealed no change in amplitude across time and between HIIE‐aerobic, HIIE‐aerobic/resistance, and seated rest. However, greater temporal stability in CDA amplitude was observed for HIIE‐aerobic compared to seated rest. These findings suggest that short bouts of HIIE may serve as an effective modality for improvements and temporal stabilization in behavior with some evidence for stabilization of neuroelectrical indices of working memory capacity. Together, these data broadly suggest that short acute bouts of exercise may facilitate improvements in underlying mental operations responsible for temporal stability in cognitive and neurocognitive function.

## Abstract 2

These data revealed that acute (9‐min) high intensity interval exercise is an effective health behavior for improving performance and temporal stability in cognitive and neuroelectrical indices of working memory capacity. These novel findings suggest that research evaluating a single “snapshot” measure may only capture a finite representation of ongoing cognitive processing, possibly limiting our understanding of temporal fluctuations in behavioral and neural indices that are impacted by acute exercise.

## 1 INTRODUCTION

Single bouts of exercise have a positive effect on cognitive function with meta‐analyses revealing potential moderators for optimal cognitive performance gains including the time window for cognitive assessment following exercise (11‐ to 20‐min), type (continuous aerobic), intensity (light‐to‐moderate), and duration of exercise (>20‐min) (Chang et al., 2012; Etnier et al., 1997; Lambourne & Tomporowski, 2010; Ludyga et al., 2016; McMorris & Hale, 2012; Verburgh et al., 2014). However, these data are complicated by recent evidence revealing similar cognitive improvements following short bouts (9‐min) of high intensity interval exercise (HIIE; Kao et al., 2017; Moreau & Chou, 2019), with additional evidence revealing sustained improvements in memory 24‐hr following the cessation of exercise (Sng et al., 2018). These recent findings suggest that the current state of the literature is far from identifying a dose–response relationship for overall cognitive outcomes (Pontifex et al., 2019), and highlight the necessity to further explore such moderators and their effects on cognitive performance following acute exercise. As such, the present investigation seeks to further elucidate exercise modality (i.e., HIIE) and timing of cognitive assessment by evaluating the effects of different HIIE modalities on the temporal dynamics of cognitive control and brain function following acute exercise in young adults.

### 1.1 Cognitive control and ERPs

Cognitive control represents a collection of goal‐directed mental operations that are responsible for coordinating top‐down processing (Botvinick et al., 2001; Norman & Shallice, 1986; Rogers & Monsell, 1995) including unified yet distinct processes of working memory, inhibitory control, and cognitive flexibility (Miyake et al., 2000). Cognitive control can further be evaluated using psychophysiological techniques including electroencephalography (EEG; summation of voltage fluctuations originating from the postsynaptic cleft measured at the scalp) and event‐related potentials (ERPs; average EEG activity time‐locked to events in the environment). ERPs provide millisecond measures of mental operations encompassing an event that may not be evident with behavior outcomes alone (Luck, 2014). Effective ERP methods require a large number of trials in order to stabilize an ERP component of interest (Huffmeijer et al., 2014). As such, ERP investigations—including acute exercise studies (Hillman et al., 2009; Kamijo et al., 2009; Kao et al., 2017, 2018; O'Leary et al., 2011; Pontifex et al., 2013; Scudder et al., 2012)—implement prolonged cognitive assessments which are then averaged across the testing interval. This prolonged “snapshot” method may be problematic for acute exercise investigations given mixed results regarding the time course for return‐to‐baseline, and evidence revealing fluctuations in cognition with moment‐to‐moment changes in mental resources for the task (Haroush et al., 2010; Kane et al., 2017). Hence, averaging across multiple prolonged assessments may only capture a finite representation of ongoing cognitive processing, possibly limiting our understanding of temporal fluctuations in behavioral and neural indices that are impacted by acute exercise. Therefore, it may be advantageous to evaluate multiple time points over a prolonged period in brain and behavior following acute exercise. This new approach may provide further insight regarding the effects of acute exercise on the stability of mental operations over time. Thus, the present investigation sought to utilize ERP methods to evaluate temporal dynamics of cognitive control—specifically working memory—and neurocognitive function following different modalities of acute HIIE.

### 1.2 Working memory, change detection task, and contralateral delay activity

Working memory is a multifaceted cognitive system that is responsible for maintaining active information in the service of updating and manipulation of that information (Baddeley & Hitch, 1974; Cowan, 1999; Kane & Engle, 2002). Recent trends have sought to evaluate capacity limits of active mental representations utilizing a visual memory change detection task (Luck & Vogel, 2013; Luria et al., 2016). This task requires individuals to remember as many items as possible in an attended array (e.g., groups of individual squares ranging in number from 2 to 8 squares) and then, after a brief retention interval, recall if any items in the attended array changed (e.g., color, orientation). This task allows for measuring individual differences in capacity limits for number of items retained in visual working memory characterized by the level when performance on the task no longer changes with increasing set size (asymptote). Interestingly, these measures of working memory capacity are positively correlated with broad measures of cognitive function and represent a unique individual difference marker for global fluid intelligence, accounting for 43% overall variance on cognitive battery measures (Fukuda et al., 2010). Hence, working memory capacity has wide‐spread implications for effective cognitive function in other domains. Regarding the present investigation, research utilizing the change detection task reveals high test–retest reliability (Dai et al., 2019) even after 1.5 years between testing sessions (Johnson et al., 2013) with further evidence demonstrating stable within‐subjects variability in performance across 30 consecutive days even when well‐practiced on the task (Xu et al., 2018). Taken together, the change detection task is well suited for the present investigation to evaluate alterations in working memory stability following acute exercise, given the robust nature of stabilization patterns observed for this measure.

The change detection task is gaining popularity in neuroimaging research given that behavior outcomes parallel neural activation during the delay retention period in both single neuron primate studies (Miller et al., 1991) and, more recently, in ERP investigations (Luck & Vogel, 2013). ERP neural activity—identified as the contralateral delay activity (CDA)—is represented as a negative slow wave over posterior electrode sites that are contralateral to the remembered items beginning ~400 ms after stimulus onset. Novel research demonstrated that CDA amplitude increases with increasing number of items to be remembered until limitations in capacity are achieved (i.e., asymptote) with further evidence demonstrating strong positive correlations of increased amplitude with better performance on the change detection task (Vogel et al., 2005; Vogel & Machizawa, 2004). Thus, it is widely accepted that CDA amplitude represents an index of capacity limits in working memory during each trial. To date, a majority of ERP investigations evaluating acute exercise effects have primarily focused on the P3 component (associated with attentional resource allocation and timing of cognitive demands) with findings revealing improvements in attentional behavior and alterations in P3 amplitude following exercise (Kao et al., 2020). Interestingly, alterations in working memory may be facilitated by attentional processes responsible for effective working memory. Although a detailed discussion of this approach is beyond the scope of the present investigation, it is worth noting that the CDA is preceded by contralateral negative activation (i.e., N2pc) that begins ~200 ms after stimulus onset and is widely used as an index of visual–spatial attention. Given that prior research reveals improvements in aspects of attentional processing and related ERP components (P3) after acute exercise, it is possible that changes in N2pc following acute HIIE may influence CDA results. Thus, the present investigation seeks to extend previous findings by incorporating a task that is well suited for generating neuroelectrical responses closely linked with working memory capacity while also investigating N2pc to determine if spatial attentional processes are influenced by acute HIIE and, in turn, possibly confound CDA outcomes.

### 1.3 High‐intensity interval exercise effects on cognition and ERPs

HIIE is characterized by short to moderate repeated bouts of high‐intensity activity (above the anaerobic threshold) intermixed with light intensity recovery periods (Weston et al., 2014). HIIE provides the same physiological health benefits as traditional continuous aerobic exercise routines accomplished in half the time (Currie et al., 2013; Gillen et al., 2016; Helgerud et al., 2007). Although limited, recent evidence suggests that HIIE may be as efficacious for cognitive benefits as continuous bouts of moderate exercise. For example, research by Kao and colleagues (Kao et al., 2017, 2018) demonstrate significant improvements in inhibitory control, memory, and reductions in P3 ERP component following 9‐ and 20‐min of aerobic HIIE (1‐min walking intervals interspersed with 1‐min high‐intensity bouts at 90% of heart rate maximum). These data—together with similar findings (Alves et al., 2014; Tsukamoto et al., 2016)—suggest that short bouts of aerobic HIIE may be as efficacious as traditional continuous exercise routines for improved cognitive and neurocognitive outcomes. However, HIIE protocols in a real‐world setting are not restricted to only aerobic intervals, and typically integrate a mix of aerobic and resistance routines including basic calisthenics that are designed as a full body high intensity strength and conditioning program. Furthermore, research suggests that this combination of aerobic and body‐weight resistance exercise is preferred in real‐world environments including work and university settings (Eather et al., 2019, 2020). Unfortunately, evidence evaluating single bouts of traditional resistance activity are mixed with data revealing both improvements (Brush et al., 2016) and no change in cognition (Chang et al., 2014; Pontifex et al., 2009) following acute bouts. These data may suggest that real‐world aerobic/resistance HIIE (HIIE‐aerobic/resistance) routines that incorporate elements of weight‐bearing resistance exercise may not be as efficacious as aerobic HIIE (HIIE‐aerobic) at improving cognitive outcomes. However, a recent investigation revealed improved performance during an attention task following HIIE‐aerobic/resistance (11‐min of body‐weight calisthenics) compared to seated rest (Walsh et al., 2018). Hence, some evidence suggests that combining modalities of resistance training with aerobic high intensity may be efficacious for improved cognitive performance. Therefore, given the necessity for research to evaluate practical applications that typically occur in real‐world settings—together with the limited findings for other cognitive control outcomes—it is necessary to further examine practical exercise modalities to better understand translational implications of acute HIIE for real‐world application.

### 1.4 Current study

The first aim of this study is to determine the temporal dynamics of working memory and CDA measures at multiple time points following acute bouts of HIIE‐aerobic and HIIE‐aerobic/resistance compared to seated rest. Previous research suggests that working memory capacity estimates are relatively stable across time which suggest a robust cognitive operation that may be immune to further changes. However, given previous acute exercise research demonstrating changes in working memory performance (Ji et al., 2017; Kamijo & Abe, 2019; Park & Etnier, 2019; Salerno et al., 2019), it was predicted that acute HIIE may follow previous research (Pontifex et al., 2019) and reveal greater benefits for trial types revealing working memory capacity (or asymptote), and have additional lasting consequences on stability for these trial types and associated ERP measures for an extended period of time beyond the acute bout. Our second aim is to evaluate HIIE‐aerobic/resistance modalities that occur in real‐world settings to determine if such methods are as efficacious as previous laboratory assessments of HIIE‐aerobic.

## 2 METHOD

### 2.1 Participants

Young adults between the age of 18 and 30 years old were recruited for the present study. Recruitment methods included announcements in Kinesiology undergraduate courses and study flyers posted throughout campus. All participants provided consent via digital signature using an online Qualtrics survey (Qualtrics, Provo, UT) in accordance with the Institutional Review Board of the University of North Carolina at Greensboro. All participants were given a unique ID to complete multiple Qualtrics surveys including health screening information, PAR‐Q, Edinburgh Handedness Inventory, and demographic information. Based on these surveys, all participants included in the study indicated that they were free of neurological or physical disabilities that prevent them from engaging in high‐intensity exercise, and had (corrected‐to‐) normal vision based on the minimal 20/20 standard. Participant data were excluded from final analyses due to dropping out after first (*n* = 2) or second (*n* = 3) laboratory visit, poor performance on the change detection task (<50% accuracy; *n* = 1), and insufficient ERP trials in one or more conditions (*n* = 2). The final analyses were performed on 22 participants (15 female; 21.4 ± 1.7 years old; 27% Black or African American; 60% White or Caucasian; 14% Alaska Native, American Indian, Asian, mixed, or other).

### 2.2 Change detection task

**FIGURE 1:**
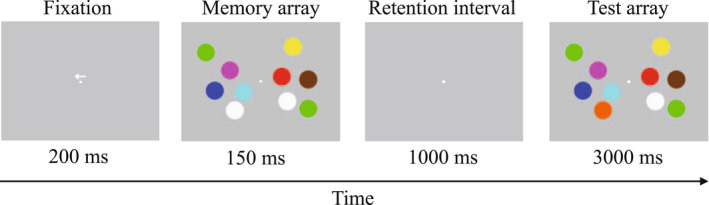
Change detection task. Example of stimulus sequence in a single trial (5‐dot set size).

The change detection task was accomplished on a computer and presented using PsychoPy presentation software with the screen at a distance of 1‐m from seated participants. As illustrated in Figure 1, a single trial included sequentially presented stimuli consisting of a direction arrow (200 ms duration), memory array (150 ms duration), retention interval (1000 ms duration), and test array (3000 ms duration). Participants were instructed to maintain visual focus on a white fixation dot presented in the center of a gray screen (RGB: 128128128) throughout each trial. While maintaining fixation, participants were directed to remember the dots in the memory array corresponding to the hemifield indicated by the direction of the arrow appearing above the fixation dot. Participants responded on a response pad during the test array by indicating whether the memory and test arrays were the same or different (“Did any of the dots in the attended hemifield change color?”). The purpose of directing attention to left and right visual hemifields is for methodological approaches for evaluating the N2pc and CDA ERP components. Responses of same or different were counterbalanced between participants with “same” responses corresponding to a left thumb press and “different” corresponding to a right thumb press for 11 participants, with the opposite response instructions given for the remaining 11 participants. Each trial consisted of random set sizes of 2, 3, 4, or 5‐dots of random color (out of a possible 10 distinct colors, RGB values: red = 255 0 0; green = 0 255 0; blue = 0 0 255; magenta = 255 0 255; yellow = 255 255 0; cyan = 0 255 255; orange = 255 103 1; white = 255 255 255; brown = 113 56 0; black = 0 0 0). Participants completed six blocks containing 80 trials each (6‐min per block; 1‐min delay between blocks; 41‐min total time to complete the task) of equiprobable set size (2‐ to 5‐dots) and detection (change or no change) that were presented equally between hemifields. Behavior and ERP result were merged across paired blocks, identified according to the time of occurrence following the intervention conditions (“40‐min” = 1st and 2nd merged blocks, “54‐min” = 3rd and 4th merged blocks, “68‐min” = 5th and 6th merged blocks), and evaluated separately to determine temporal changes in brain function and task performance following the treatment conditions. Calculation of individual memory capacity followed previous research [*k* = (hit rate—false alarm rate) × set size] such that *k* is assumed to increase with set size load and reach asymptote at an individual’s working memory capacity (Cowan, 2001).

### 2.3 ERP recordings

EEG activity was recorded from 64 Ag/AgCl sintered electrode sites arranged in an extended montage according to the International 10–10 system (Chatrian et al., 1985) using a Neuroscan Quik‐Cap (Compumedics, Inc., Charlotte, NC) with AFz serving as the ground electrode. Additional bipolar electrodes were placed to monitor vertical and horizontal electrooculographic (EOG) activity positioned above and below the left orbit and on the outer canthus of both eyes. Prior to EEG recordings, electrode impedance was maintained at <10 kΩ. Continuous data were referenced to a midline electrode placed at midpoint between Cz and CPz and digitized at a sampling rate of 1000 Hz and amplified 500 times with a DC to 70 Hz filter using a Neuroscan SynAmps RT amplifier. Offline, data were processed utilizing MATLAB (R2019) and various toolbox plug‐ins including EEGLAB (Delorme & Makeig, 2004) and ERPLAB (Lopez‐Calderon & Luck, 2014). Data were re‐referenced to average mastoids (M1, M2) and high‐pass filtered (0.1 Hz). Eyeblink artifact was removed utilizing an automated independent component analyses (ICA) procedure. ICA decompositions were performed using the extended infomax algorithm followed by a time series correlation method that compared point‐by‐point raw VEOG data with separate ICA activation waveforms (i.e., EEG.icaact matrix generated by the ICA procedure). ICA factors were removed if the correlation coefficient was greater than 0.30. Data were then back projected without rejected ICA components and visual checked to determine attenuation of eyeblink artifact.

Stimulus‐locked epochs were extracted (−200 ms to 1000 ms; baseline corrected, and low‐pass filtered at 30 Hz) for each set size during the retention interval if a correct response occurred during the test array. Epochs were rejected if a moving window peak‐to‐peak amplitude exceeded 100 μV (100‐ms window width and 50‐ms window step), if the overall variance of the epoch exceeded ±3 SDs of the mean of local (by electrode site) and global (all electrode sites) accepted epochs, and by visual inspection of HEOG channels for stereo‐typical horizontal eye movement. CDA was determined by subtracting contralateral (opposite hemifield of attended array) electrode activity from ipsilateral activity for each set size. Recent research indicates a minimum of 30–50 clean trials to detect the presence of a CDA (i.e., reliable difference between contralateral and ipsilateral) and 400 clean trials to detect differences in CDA amplitude between two set sizes (Ngiam et al., 2021). Given the limited trials to detect differences between set sizes in the present study (120 total trials per set size), CDA waveforms were created by averaging 100 ± 15 epochs across all set sizes separately within each time interval (i.e., 40‐min, 54‐min, 68‐min). Analysis of CDA mean amplitude (300 ms to 1000 ms) and N2pc mean amplitude (230 ms to 300 ms) were performed at electrode sites TP7/TP8, CP5/CP6, P7/P8, P5/P6, PO7/PO8, and PO5/PO6.

### 2.4 Procedure

Using a within‐participants cross‐over design, all participants visited the laboratory on four separate days. Participants were instructed to avoid vigorous physical activity and to maintain typical daily behaviors (i.e., sleep, food and beverage consumption, work/school activities) 12 h prior to testing. Time of day for all visits to the laboratory was held constant such that each participant completed all sessions at the same time on separate days. During the first visit, participants reviewed the informed consent with the experimenter, completed practice trials for the cognitive task, and performed a cardiorespiratory fitness assessment on a motor driven treadmill following a modified Balke Protocol (American College of Sports Medicine, 2014). The purpose of the fitness assessment was to obtain maximum heart rate (HR) measures that aid in determining individualized exercise intensity levels during the experimental conditions. Maximal aerobic capacity (VO_2max_) was measured using a computerized indirect calorimetry system (ParvoMedics True Max 2400) and determined based on ACSM guidelines (American College of Sports Medicine, 2014) and criteria for successfully achieving maximal output in adults.

The three experimental conditions were counterbalanced and administered randomly across participants to minimizing practice/learning effects. Each condition consisted of 9‐min of HIIE‐aerobic performed on a treadmill, HIIE‐aerobic/resistance calisthenics, and seated rest while watching an educational video. The HIIE‐aerobic condition replicates prior work (Kao et al., 2017) such that participants warmed‐up on a treadmill for 1‐min (60% HR_max_) followed by three repetitions of 1.5 min running (increased speed and incline to achieve 90% HR_max_) with intermittent 1‐min walking bouts (decrease speed and incline to achieve 60%–70% HR_max_) and a final cool‐down walking period of 1.5 min (<60% HR_max_). During the HIIE‐aerobic/resistance condition, participants followed the same timing protocol as HIIE‐aerobic except for the type of exercise during the 1.5 min at 90% HR_max_. Participants completed repeated rounds of 10‐m shuttle run, 20 jumping jacks, 10‐m skipping, 15 air squats, 20 high knees, and 10‐m walking lunges (90% HR_max_) within each 1.5‐min bout. The HIIE‐aerobic/resistance protocol was developed based on prior feasibility research evaluating real‐world HIIE protocols in natural work and education environments (Eather et al., 2019, 2020), and recommendations from certified personal trainers in the community. The intent was to evaluate a previously tested lab‐based protocol (HIIE‐aerobic; Kao et al., 2017) to a method that has translational potential for real‐world application. For the rest condition, participants viewed an emotionally neutral educational video (*Join this Man on a Safari to Sculpt Animals in the Wild; National Geographic*). A Polar HR monitor (Model H7, Polar Electro, Finland) was used to measure HR throughout each condition that correspond with transition intervals during the HIIE conditions from moderate‐ to high‐intensity (1‐min, 3.5‐min, 6‐min), from high‐ to moderate‐intensity (2.5‐min, 5‐min, 7.5‐min), and at the cessation of the exercise conditions (9‐min). Following the treatment conditions, participants were fitted with an EEG cap and seated in a quiet testing chamber where the working memory change detection task was completed. Time between cessation of treatment conditions to start of the change detection task was held constant at 40‐min while the working memory task occurred between 40‐min (start of cognitive testing) to 81‐min (end of cognitive testing). This specific time interval to measure working memory was determined based on (1) the time delay necessary to put on an EEG cap, and (2) a priori based on previous literature in an effort to capture the return‐to‐baseline period following acute exercise (Chang et al., 2012). Although the exact timing of persistent effects is not clear (Pontifex et al., 2019), evaluation of trends in the literature informed our approach.

### 2.5 Statistical analysis

Statistical procedures were conducted using SPSS (v.26, SPSS, Chicago, IL). Repeated measures ANOVAs were performed with findings reported using Huynh‐Feldt correction statistic for violations of sphericity. Reporting of main effects and interactions included partial *η* ^2^. Post hoc *t* test comparisons included reporting of estimated effect size for repeated measures (Cohen’s *d*; small *d* = 0.2, medium *d* = 0.5, and large *d* = 0.8 effect sizes) with false discovery rate correction [FDR; (individual *p* value rank/total number of comparisons)*false discovery rate i.e., 0.15] (Benjamini & Hochberg, 1995) used to determine significance level for multiple comparisons. Additionally, a sensitivity power analysis was performed (G*Power v3.1.9; Faul et al., 2007) to determine the strength and reliability of an effect size in the current data set with a sample size of *n* = 22. With an alpha level = .05 and power = .80, the minimum effect size that is estimated to reliably detected differences in a within subject *t* test is *d* = 0.5 (i.e., medium to large effect size). Cognitive task *k*‐score accuracy was analyzed utilizing a 3 (Mode: HIIE‐aerobic/resistance, HIIE‐aerobic, seated rest) × 3 (Time: 40‐min, 54‐min, 68‐min) × 4 (Size: 2‐dot, 3‐dot, 4‐dot, 5‐dot) model. CDA and N2pc amplitude were analyzed separately utilizing a 3 (Mode: HIIE‐aerobic/resistance, HIIE‐aerobic, seated rest) × 3 (Time: 40‐min, 54‐min, 68‐min) × 6 (Site: TP7/TP8, CP5/CP6, P7/P8, P5/P6, PO7/PO8, PO5/PO6) model. Methods for calculating temporal stability were adopted from previous research evaluating the change detection task (Xu et al., 2018) and was accomplished by calculating correlation coefficients and reliability intraclass correlation (ICC) from a two‐way mixed effects model (absolute agreement definition) to provide an index of agreement for temporally adjacent sessions (i.e., 40‐min with 54‐min, 54‐min with 68‐min) for behavior and ERP measures. Reliability was determined as poor (<0.5), moderate (0.5 to 0.75), good (0.75 to 0.9), and excellent (>0.9).

## 3 RESULTS

**FIGURE 2:**
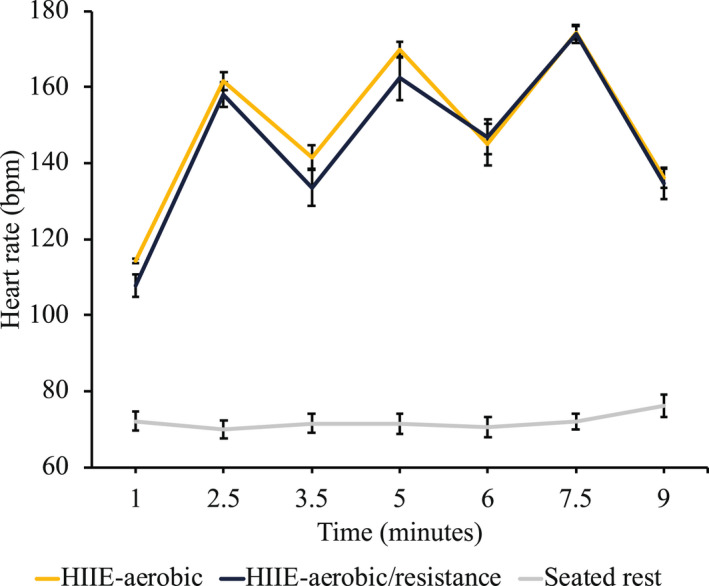
Heart rate during intervention. Heart rate (measured in beats per minute) by treatment condition at time intervals that correspond to transitions from moderate‐ to high‐intensity, and from high‐ to moderate‐intensity.

Analysis with session order as a between‐subjects factor for behavior and ERP data revealed no significant interactions with outcome results reported below, *F*’s (18.9, 179.9) ≤ 0.74, *p*’s ≥ .77, $\eta_{p}^{2}$ ≤ .07. ERP analysis of trial count revealed no Mode interactions, *F*’s (2.6, 55.9) ≤ 1.64, *p*’s ≥ .20, $\eta_{p}^{2}$ ≤ .07, suggesting consistent trial count between treatment conditions across time. Lastly, HR analysis revealed greater average HR for both HIIE conditions (HIIE‐aerobic = 149.0 ± 1.9 bpm; HIIE‐aerobic/resistance = 145.4 ± 2.9 bpm) compared to seated rest (72.0 ± 2.4) across all time points, *t*’s ≥ 3.23, *p*’s ≤ .05, with no difference observed between HIIE conditions, *t*’s ≤ 2.10, *p*’s ≥ .06 (see Figure 2).

### 3.1 Change detection task results

The omnibus analysis for *k* score accuracy revealed a main effect of Size, *F* (1.4, 28.9) = 88.67, *p* ≤ .01, $\eta_{p}^{2}$ = .81, revealing increasing *k* score accuracy with increasing dot size, *t*’s (21) ≥ 5.43, *p*’s ≤ .01, *d*’s ≥ 1.16, with asymptote occurring between 4‐dot (3.1 ± 0.1 *k*‐score) and 5‐dot accuracy (3.2 ± 0.2 *k*‐score), *t* (21) = 1.28, *p* = .21. Additionally, analysis revealed a main effect of Time, *F* (1.9, 40.5) = 6.69, *p* ≤ .01, $\eta_{p}^{2}$ = .24, and an interaction of Time × Size, *F* (3.7, 78.2) = 2.93, *p* = .03, $\eta_{p}^{2}$ = .12, that were all superseded by a Mode × Time × Size interaction, *F* (8.3, 175.8) = 2.21, *p* = .03, $\eta_{p}^{2}$ = .10. Decomposition comparing treatment conditions within each Time interval revealed greater 4‐dot *k* score accuracy at 40‐min for HIIE‐aerobic/resistance (3.4 ± 0.1 *k*‐score) compared to seated rest (3.0 ± 0.1 *k*‐score), *t* (21) = 3.44, *p* ≤ .01, *d* = 0.74. No other main effects or interactions between treatment conditions were observed in the 54‐min and 68‐min interval, *F*’s (4.5, 95.6) ≤ 2.20, *p*’s ≥ .07, $\eta_{p}^{2}$ ≤ .10. Decomposition of the interaction across Time within each set size revealed greater 5‐dot *k* score accuracy for HIIE‐aerobic/resistance in the 40‐min interval (3.6 ± 0.2 *k*‐score) compared to the 54‐min (3.2 ± 0.2 *k*‐score) and 68‐min intervals (3.1 ± 0.2 *k*‐score), *t*’s (21) ≥ 3.26, *p*’s ≤ .01, *d*’s ≥ 0.60. Further, seated rest revealed greater 5‐dot *k* score accuracy in the 40‐min (3.3 ± 0.2 *k*‐score) and 54‐min (3.4 ± 0.2 *k*‐score) intervals compared to the 68‐min interval (2.9 ± 0.2 *k*‐score), *t*’s (21) ≥ 2.64, *p*’s ≤ .01, *d*’s ≥ 0.55 (see Table 1). Lastly, between‐session temporal stability revealed greater average Pearson correlations and ICC reliability across temporally adjacent sessions for HIIE‐aerobic/resistance (average across all dot sizes, *r*_mean_ = 0.67, ICC_mean_ = 0.78) and HIIE‐aerobic (*r*_mean_ = 0.73, ICC_mean_ = 0.82) compared to seated rest (*r*_mean_ = 0.46, ICC_mean_ = 0.57; see Table 2).

**TABLE 1:** Mean (±*SEM*) of change detection results

Measure	Seated rest			HIIE‐aerobic/resistance			HIIE‐aerobic		
	40‐min	54‐min	68‐min	40‐min	54‐min	68‐min	40‐min	54‐min	68‐min
2‐dot *k*‐score	1.9 ± 0.0	1.8 ± 0.1	1.9 ± 0.0	1.9 ± 0.0	1.9 ± 0.0	1.9 ± 0.0	1.9 ± 0.0	1.9 ± 0.0	1.8 ± 0.0
3‐dot *k*‐score	2.8 ± 0.1	2.7 ± 0.1	2.6 ± 0.1	2.7 ± 0.0	2.6 ± 0.1	2.6 ± 0.1	2.6 ± 0.1	2.5 ± 0.1	2.6 ± 0.1
4‐dot *k*‐score	3.0 ± 0.1	3.1 ± 0.2	3.0 ± 0.2	3.4 ± 0.1	3.2 ± 0.1	3.2 ± 0.1	3.2 ± 0.1	3.1 ± 0.1	3.1 ± 0.1
5‐dot *k*‐score	3.3 ± 0.2	3.4 ± 0.2	2.9 ± 0.2	3.6 ± 0.2	3.2 ± 0.2	3.1 ± 0.2	3.3 ± 0.2	3.2 ± 0.2	3.0 ± 0.2

**TABLE 2:** Person correlation and inter‐class correlation (ICC) reliability coefficient summary table for temporally adjacent change detection task performance and ERP amplitude for N2pc and CDA

Measure	Seated rest				HIIE‐aerobic/resistance				HIIE‐aerobic			
	40‐min to 54‐min		54‐min to 68‐min		40‐min to 54‐min		54‐min to 68‐min		40‐min to 54‐min		54‐min to 68‐min	
Statistic	ICC	Person	ICC	Pearson	ICC	Pearson	ICC	Pearson	ICC	Pearson	ICC	Pearson
2‐dot *k*‐score	.20	.22	.37	.24	.64^a^	.46^b^	.65^a^	.48^b^	.74^a^	.58^a^	.90^a^	.83^a^
3‐dot *k*‐score	.59^b^	.43^b^	.52^b^	.35	.63^a^	.57^a^	.90^a^	.82^a^	.80^a^	.72^a^	.79^a^	.68^a^
4‐dot *k*‐score	.71^a^	.56^a^	.73^a^	.58^a^	.81^a^	.71^a^	.91^a^	.82^a^	.77^a^	.64^a^	.82^a^	.69^a^
5‐dot *k*‐score	.82^a^	.72^a^	.64^a^	.58^a^	.78^a^	.70^a^	.89^a^	.80^a^	.88^a^	.80^a^	.93^a^	.88^a^
CDA (μV)	.80^a^	.70^a^	.85^a^	.74^a^	.72^a^	.59^a^	.76^a^	.62^a^	.90^a^	.85^a^	.77^a^	.63^a^
N2pc (μV)	.83^a^	.79^a^	.81^a^	.73^a^	.78^a^	.64^a^	.84^a^	.73^a^	.85^a^	.74^a^	.80^a^	.67^a^

^a^ Significant at the .01 level (two‐tailed).

^b^ Significant at the .05 level (two‐tailed).

### 3.2 ERP results

**FIGURE 3:**
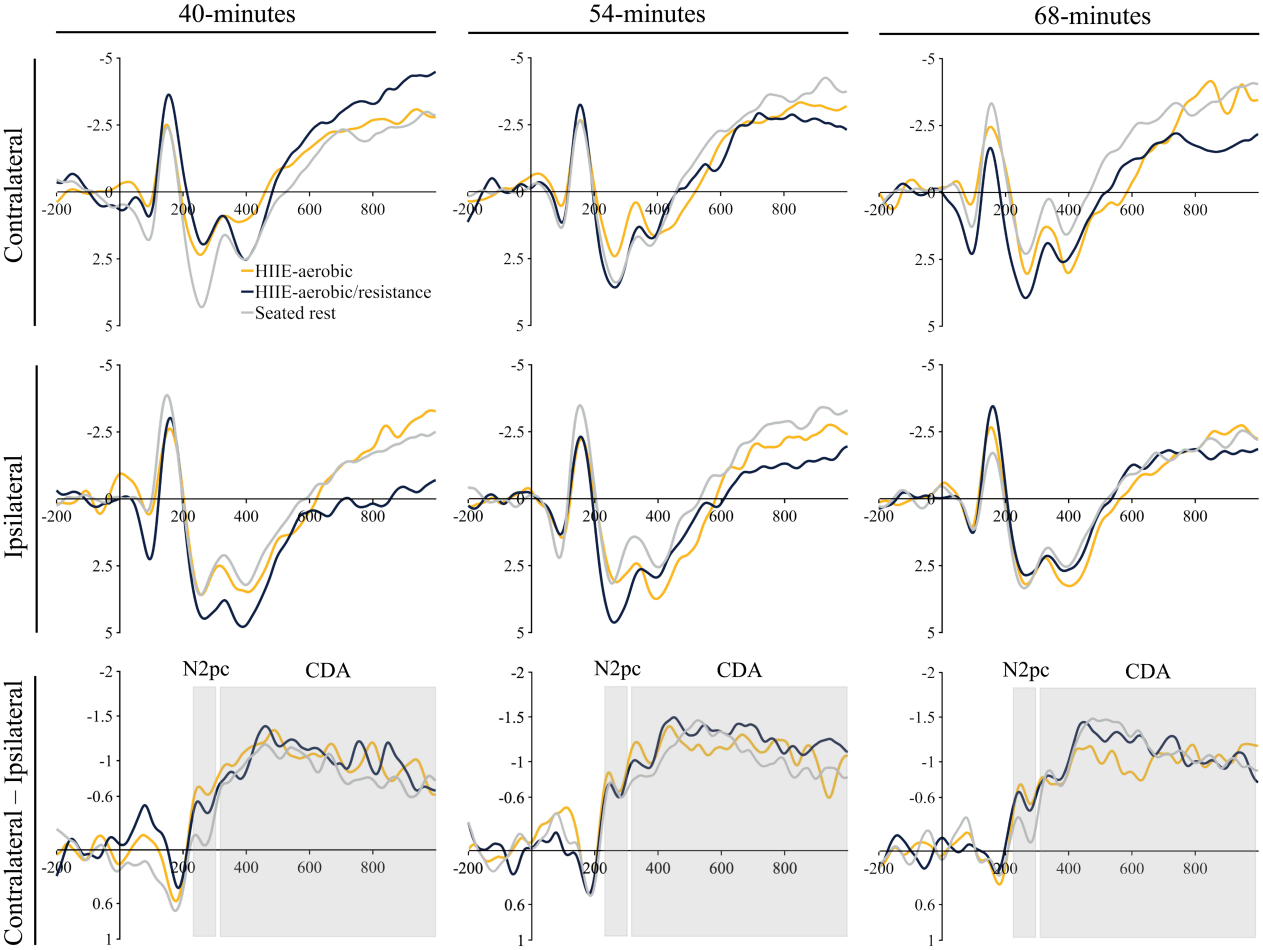
ERP waveforms. Grand average ERP waveforms (averaged across sensors TP7/TP8, CP5/CP6, P7/P8, P5/P6, PO7/PO8, and PO5/PO6) separated by treatment conditions of HIIE‐aerobic, HIIE‐aerobic/resistance, and seated rest for contralateral (top row), ipsilateral (middle row), and contralateral‐ipsilateral differences waves (bottom row) for each time interval (columns). Gray bands indicate the measurement window for assessing the N2pc and CDA mean amplitude. All waveforms were low‐pass filtered (20 Hz) for visual clarity.

**FIGURE 4:**
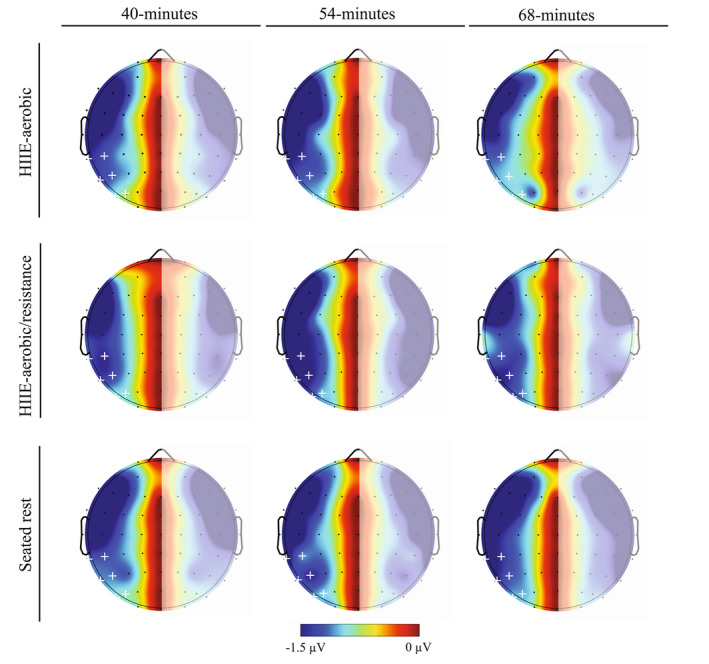
CDA topographic plots. Topographic maps of CDA mean amplitude (300 ms to 1000 ms) representing contralateral—ipsilateral activity with mirrored representation across left and right (faded area) hemifields. White plus symbols (+) indicate electrodes included in the analyses (TP7/TP8, CP5/CP6, P7/P8, P5/P6, PO7/PO8, and PO5/PO6).

The omnibus analysis for N2pc revealed no main effects or interactions, *F*’s ≤ 2.90, *p*’s ≥ .08, $\eta_{p}^{2}$’s ≤ .12. The omnibus analysis for CDA revealed a main effect of site, *F* (2.0, 41.6) = 5.64, *p* < .01, $\eta_{p}^{2}$ = .21, revealing a general trend of smaller amplitude for parietal‐occipital electrode sites (PO7/PO8 = −0.87 ± 0.22 μV; PO5/PO6 = −0.79 ± 0.21 μV) compared to the remaining electrode sites (i.e., TP7/TP8 = −1.27 ± 0.15 μV; CP5/CP6 = −1.14 ± 0.14 μV; P7/P8 = −1.13 ± 0.19 μV; P5/P6 = −1.10 ± 0.19 μV), *t*’s (21) ≥ 2.45, *p*’s ≤ .02, *d*’s ≥ 0.67. No other main effects or interactions were revealed, *F*’s ≤ 2.05, *p*’s ≥ .14, $\eta_{p}^{2}$’s ≤ .09 (see Figures 3 and 4). Lastly, between‐session stability for CDA revealed “excellent” reliability for the HIIE‐aerobic condition between 40‐ and 54‐min interval (ICC = .90, *r* = .85) while the remaining results for N2pc and CDA across time and condition revealed “moderate” to “good” reliability (see Table 2).

## 4 DISCUSSION

The purpose the present study was to investigate temporal dynamics of working memory and associated neural responses following different modalities of HIIE‐aerobic/resistance and HIIE‐aerobic compared to seated rest. The main behavioral findings revealed that both HIIE conditions facilitate improvements in working memory capacity and demonstrates persistent stabilizing behavior patterns across repeated measures to a greater degree compared to seated rest. Additionally, ERP results revealed greater temporal stabilizing patterns for CDA amplitude during the HIIE‐aerobic condition between the 40‐ and 54‐min time interval. Together, these findings suggest that short bouts of HIIE may be more efficacious for improving and stabilizing behavioral and neural indices of working memory across time compared to inactivity bouts.

The behavior results in the present study are in‐line with previous findings suggesting greater acute exercise effects for more difficult trial conditions (Pontifex et al., 2019). Here, asymptote occurred between 4‐ and 5‐dot accuracy—suggesting capacity limits in working memory load for the more difficult trial conditions—with acute effects revealing greater improvements for 4‐dot working memory load selective for the HIIE‐aerobic/resistance condition. Furthermore, these improvements were only observed during the 40‐min period, returning to a level comparable to seated rest and HIIE‐aerobic at 54‐min and 68‐min. These data suggest that acute HIIE‐aerobic/resistance effects on working memory persist for a short interval post exercise and return‐to‐baseline approximately 1‐hr after the exercise bout. However, results of temporal changes and stability may shed additional light on persistent beneficial effects beyond this period. Specifically, HIIE‐aerobic revealed stable performance across each time interval with no change in performance across all set sizes, while the HIIE‐aerobic/resistance and seated rest condition revealed significant reductions in 5‐dot performance across time. Further, ICC reliability and correlation results revealed a noticeable improvement in stability for both HIIE conditions compared to seated rest for all trial types including set sizes of low working memory load trials (2‐dot and 3‐dot set size) suggesting that acute bouts of HIIE provide a global benefit for stabilizing temporal working memory performance across an extended period. Together, these data provide novel evidence suggesting that acute exercise may impact processes that improve and regulate temporal stability in mental operations necessary for consistent working memory performance across time.

Examination of the CDA and N2pc component revealed no differences in amplitude between conditions and across time. However, stability analysis for CDA amplitude revealed greater temporal stability for the HIIE‐aerobic condition (i.e., ICC revealed excellent reliability) between 40‐ and 54‐min time interval compared to HIIE‐aerobic/resistance and seated rest (i.e., ICC revealed good reliability). CDA is a neurophysiological marker of between and within variation in working memory storage and maintenance (Luria et al., 2016; Vogel et al., 2005), with amplitude indicative of successful storage of items in working memory (Adam et al., 2018). Hence, these data suggest that short acute bouts of HIIE‐aerobic may serve as an effective method for stabilizing mechanisms necessary for successfully storing items in working memory across an extended interval post exercise. Additionally, the null results for the N2pc suggest that neuroelectrical markers of visual–spatial attention are not influenced by acute bouts of HIIE, further supporting the conclusion that observed temporal stability for CDA amplitude are not confounded by the N2pc.

Together, the behavior and ERP results support the first aim and demonstrate significant temporal benefits of short bouts of HIIE on behavioral measures of working memory and supporting neuroelectrical underpinnings. However, the second aim was only partially supported revealing selective and mutual modality benefits for HIIE‐aerobic and HIIE‐aerobic/resistance methods typically observed in real‐world settings. That is, these data suggest that engaging in aerobic or aerobic‐resistance calisthenics when accomplishing short bouts of HIIE will benefit working memory outcomes in a similar manner. However, it is not clear from the present data if exercise modality moderates working memory and associated ERP components given no clear benefits across conditions. One possibility may be that both HIIE‐aerobic and HIIE‐aerobic/resistance conditions maintained the same intensity level (i.e., no difference in HR). Research suggests that intensity may be a determining factor regarding the after‐effects on cognition (Chang et al., 2012). This is evident in previous HIIE research revealing positive benefits for inhibitory control outcomes for both continuous moderate‐intensity exercise and HIIE‐aerobic with greater magnitude of benefits for the more intense HIIE condition (Kao et al., 2018). Regardless, although there was no clear distinction of outcomes between the two conditions, the present findings do suggest that benefits to working memory are evident for HIIE of a similar intensity level regardless of type of exercise routine.

### 4.1 Mechanisms

Recent models of cognitive control may shed light on possible mechanisms related to the present findings. Specifically, these models suggest that efficient cognitive control performance is dependent on two independent networks that operate in parallel to accomplish appropriate behavioral control (Bressler & Menon, 2010; Menon & Uddin, 2010). The first network is responsible for regulation of mental operations on a trial‐by‐trial basis with greater brain activation occurring in the frontoparietal region during cognitive control tasks. The second network reveals activation in the cingulo‐opercular region and is related to stability and maintenance of goal‐directed behavior throughout performance (Cocchi et al., 2013; Dosenbach et al., 2007; Dosenbach et al., 2008). This stability network works to regulate task performance and control goal‐directed behavior. Accordingly, given the differences in temporal stability between rest and acute HIIE in the present investigation, it appears that HIIE may influence both networks with additional evidence for improved stability patterns in cognitive performance across time (Haroush et al., 2010; Kane et al., 2017). As such, it may be critical for future investigations to evaluate not only overall performance in cognition and neurophysiological outcomes at a single interval, but also to include multiple measures across time to further elucidate beneficial effects for mechanisms responsible for stability of behavior and underlying brain function patterns.

### 4.2 Limitations

There are several limitations worth noting. First, the present investigation did not include a pretest measurement immediately prior to the exercise and rest sessions. The inclusion of a baseline measure reduces the impact of day‐to‐day variation in factors that may influence working memory and CDA measures. However, including a pretest condition might increase subject burden as the current study design took approximately 2‐hr to complete for each testing visit. Next, there were no assessments of working memory and CDA at early time points between the cessation of HIIE and the start of the change detection task. Previous investigations reveal improved cognitive control performance immediately following and up to 30‐min post HIIE (Alves et al., 2014; Hwang et al., 2017; Kao et al., 2017, 2018; Tsukamoto et al., 2016; Walsh et al., 2018) with no research to date evaluating HIIE effects past 30‐min. Although this window of time was not assessed, the novelty of the present investigation measuring beyond this interval reveals that acute effects of short bouts of HIIE return to a level comparable to seated rest within 1 hr with extended benefits in stability that remain for an unknown time. Future research should seek to incorporate more frequent assessments at earlier and extended intervals to further determine time points of peak benefits and return‐to‐baseline. Lastly, although the present study incorporated HIIE methods that translate into real‐world settings, the present study was accomplished in a controlled laboratory space. Future research might benefit by having participants engage in HIIE accomplished in their own environment. With advancements in EEG technology it is now possible to capture both cognition and EEG outcomes using mobile and wireless devices. This new direction may further advance our understanding of how laboratory findings match real‐world exercise experience and the impact on mental health.

## 5 CONCLUSION

These data suggest that acute bouts of HIIE improve working memory behavioral and neuroelectric responses for an extended period beyond the cessation of the exercise bout. Although distinct differences between HIIE conditions were not observed, this new approach of evaluating temporal dynamics may shed light on recent findings that demonstrate sustained improvements in cognitive performance even after a delay of 24‐hr following the cessation of the exercise bout. Given that cognitive processing of information is subject to unaccounted interference that may change performance from moment‐to‐moment, it is possible that previous investigations fall short by only comparing cognitive outcomes at a single time point. The present findings are novel and shed new light for future investigations suggesting that cognitive and neuroelectrical outcomes may be revealed by multiple assessments over time that will help inform our understanding of temporal dynamics of brain and behavior that extend beyond a “snapshot” of our everyday functioning.

## AUTHOR CONTRIBUTIONS

**Eric S. Drollette:** Conceptualization; data curation; formal analysis; investigation; methodology; project administration; resources; supervision; writing – original draft. **Caroline C. Meadows:** Data curation; writing – review and editing.

## CONFLICT OF INTEREST

No conflicting financial interests exist.
